# Improved reference quality genome sequence of the plastic-degrading greater wax moth, *Galleria mellonella*

**DOI:** 10.1093/g3journal/jkae070

**Published:** 2024-04-02

**Authors:** Reginald Young, Khandaker Asif Ahmed, Leon Court, Cynthia Castro-Vargas, Anna Marcora, Joseph Boctor, Cate Paull, Gene Wijffels, Rahul Rane, Owain Edwards, Tom Walsh, Gunjan Pandey

**Affiliations:** CSIRO Environment, Acton, ACT 2601, Australia; CSIRO Australian Animal Health Laboratory, Geelong, VIC 3220, Australia; CSIRO Environment, Acton, ACT 2601, Australia; CSIRO Environment, Acton, ACT 2601, Australia; CSIRO Agriculture and Food, Dutton Park, QLD 4102, Australia; Bioplastics Innovation Hub, Food Futures Institute, Murdoch University, Murdoch, WA 6150, Australia; CSIRO Agriculture and Food, Dutton Park, QLD 4102, Australia; CSIRO Agriculture and Food, St Lucia, QLD 4067, Australia; CSIRO Health and Biosecurity, Parkville, VIC 3052, Australia; CSIRO Environment, Floreat, WA 6010, Australia; CSIRO Environment, Acton, ACT 2601, Australia; CSIRO Environment, Acton, ACT 2601, Australia

**Keywords:** bioremediation, genome assembly, plastic degradation, polyethylene, secretory proteins, wax moth

## Abstract

*Galleria mellonella* is a pest of honeybees in many countries because its larvae feed on beeswax. However, *G. mellonella* larvae can also eat various plastics, including polyethylene, polystyrene, and polypropylene, and therefore, the species is garnering increasing interest as a tool for plastic biodegradation research. This paper presents an improved genome (99.3% completed lepidoptera_odb10 BUSCO; genome mode) for *G. mellonella*. This 472 Mb genome is in 221 contigs with an N50 of 6.4 Mb and contains 13,604 protein-coding genes. Genes that code for known and putative polyethylene-degrading enzymes and their similarity to proteins found in other Lepidoptera are highlighted. An analysis of secretory proteins more likely to be involved in the plastic catabolic process has also been carried out.

## Teaser

The greater wax moth, *Galleria mellonella*, has recently garnered interest as an organism capable of plastic degradation. In this work, Young et al. have assembled a high quality genome for the greater wax moth to uncover the key enzymes housed in the larvae of these insects. Using their assembly, the authors have identified several candidate plastic-degrading proteins.

## Introduction

The greater wax moth, *Galleria mellonella*, is a common pest of honeybees because its larvae feed on beeswax and other components of honeybee hives (Kwadha *et al*. 2017). Recently published literature has confirmed the ability of wax moth larvae to catabolize low-density polyethylene (PE) at higher rates than have yet to be observed in other organisms (Sanluis-Verdes *et al*. 2022). This ability of the wax moth larvae to break down such a recalcitrant material may be attributed to its structural similarity to the long-chain hydrocarbon compounds found in beeswax (Fratini *et al*. 2016). There is also evidence of the ability of the larvae to degrade other commercially produced plastics such as polystyrene and polypropylene (Lou *et al*. 2022; Ruiz Barrionuevo *et al*. 2022). As a result, the species is garnering increasing interest as a tool for plastic bioremediation (An *et al*. 2023).

Both the wax moth larvae themselves and their gut microbiome have been implicated in the degradation of PE in different studies. Some studies have shown evidence that suppression of the microbiota results in decreased degradation, while others have shown that the wax moth larvae can break down certain plastics axenically (Cassone *et al*. 2020; Lou *et al*. 2020; Rejasse *et al*. 2022; Wang *et al*. 2022). Kong *et al*. (2019) showed that wax moth larvae successfully degrade or hydrolyze long-chain hydrocarbons from wax independently of the gut microbiota, but the latter acts as a secondary degrader after long-chain fatty acid production (Kong *et al*. 2019).

Recently, several enzymes from the saliva of the larvae of the greater wax moth have been shown to possess PE-degrading capabilities (Sanluis-Verdes *et al*. 2022; Spínola-Amilibia *et al*. 2023). These PE-degrading enzymes are hexamerin and arylphorin proteins, which are a part of the hemocyanin superfamily and exist as large hexameric complexes. Interestingly, hexamerins and arylphorins generally perform nonenzymatic roles in early and nonfeeding stages of insects (Tian *et al*. 2021), acting primarily as storage proteins (Martins and Bitondi 2016; Dutta *et al*. 2020). The nonconventional activity exhibited among these *G. mellonella* salivary proteins suggests that the organism may have evolved these proteins to develop long-chain hydrocarbon-degrading capabilities. Three of these proteins, Demetra, Ceres, and Cora, can each oxidize PE, and a fourth, Cibeles, acts as a partner protein for Demetra in a hexameric complex but does not exhibit PE-degrading activity by itself. The discovery of these enzymes presents a significant step toward bioremediation of PE, which has been the plastic with the highest global annual production for several decades (Yao *et al*. 2022).

The availability of recent draft genome sequences (Lange *et al*. 2018; Kong *et al*. 2019) has provided insights into the role of the wax moth in PE pollutant degradation. An initial 578.5 Mb wax moth draft genome (GCA 002589825.1; 1,937 contigs) was made available in 2018 (Lange *et al*. 2018), with 2 additional 483.2 and 401.9 Mb assemblies (GCA 004355975.1; GCA 003640425.2) published in 2019 (Kong *et al*. 2019), the latter of which was the previous reference genome. As the previous RefSeq was quite fragmented, we aimed to construct a more contiguous genome that covered more of the lepidopteran genes with a combination of long- and short-read sequencing data. The improved genome presented here sheds new light on the mechanisms by which the wax moth breaks down various plastics.

## Materials and methods

### Insect collection

*Galleria mellonella* was cultured in controlled environment rooms at the CSIRO Insect Laboratory, Queensland Ecosciences Precinct, Dutton Park, Queensland, Australia. To establish the *G. mellonella* culture, larvae were collected from an infested beehive in suburban Brisbane, Australia, on 2021 June 2. The larvae were maintained on a diet of retail grade yeast, honey, glycerol, and baby cereal (Farex). Environmental conditions included a 14:10 h light–dark cycle with a constant temperature of 26°C and 50% relative humidity. Five healthy adults successfully mated and yielded viable eggs and larvae. The specimen sequenced was from the second generation in laboratory culture. Note that the sex of the specimen was not able to be determined at the larval stage.

### DNA extraction

High-molecular-weight DNA was extracted using the Qiagen Genomic Tip 20/G kit (Qiagen, Cat#: 10223) and the Qiagen Buffer Set (Qiagen, Cat#: 19060). The “User-developed protocol” for “mosquitoes and other insects” supplied by Qiagen was used, although Qiagen elution buffer (EB) was used to dissolve the purified genomic DNA instead of Tris-EDTA buffer.

### Genome sequencing

For Illumina short-read sequencing, Illumina PCR-based libraries were commercially constructed as per the manufacturer's protocol (Azenta-China, Suzhou facility) and sequenced to generate 32.54 Gb (108.46 million raw reads) on an Illumina NovaSeq 6000 sequencer, S4 flow cell lane (2 × 150 bp PE). Short-read sequencing libraries were constructed using a NEBNext Ultra II DNA Library Prep kit for Illumina (NEB, USA) and sequenced using the HiSeq X platform (Illumina, Inc, San Diego, CA, USA) run in 150-bp paired-end mode.

For Oxford Nanopore Technology (ONT) long-read sequencing, shorter DNA fragments (<5 kb) were depleted from the *G. mellonella* DNA extract using the Circulomics SRE XS protocol (Circulomics, Cat# SS-100-121-01). The size-selected genomic DNA was then prepared for sequencing using the ONT native barcoding workflow and then sequenced on an ONT PromethION Sequencer at the Biomolecular Resource Facility at the John Curtin School of Medical Research, Australian National University. Long-read sequencing was carried out on both MinION and PromethION platforms, using a library constructed with an SQK-LSK109 kit (Oxford Nanopore, UK) in both cases and FLO-MIN106D and FLO-PRO002 flow cells, respectively.

### Genome assembly

Adapter sequences of the short-read sequencing data were removed from the resulting reads using TrimGalore v0.6.6 (Krueger 2015). Base calling on the raw ONT long reads was performed using guppy v5.1 (Wick *et al*. 2019) under the dna_r9.4.1_450bps_sup.cfg model. The long-read sequences were trimmed using Porechop v0.2.4 (Wick *et al*. 2017). The long reads were assembled using SMARTdenovo v1 (Kolmogorov *et al*. 2020) with a Smith–Waterman free dot matrix alignment (-e dmo), kmer size of 16 (-k 16), and minimum read length of 1 kb (-J 1000). The resulting genome assembly was then subjected to 3 rounds of polishing using long reads and 3 rounds using short reads with Racon v1.4.22 (Vaser *et al*. 2017) under default settings. This was followed by 3 rounds of short-read-based polishing using Polca MaSURCA v4.0.7 (Zimin *et al*. 2013) with default settings to obtain the final contig assembly. This assembly was assessed using Benchmarking Universal Single-Copy Orthologs (BUSCO) v5.2.2 (Simão *et al*. 2015) against the Insecta lineages' gene sets (insecta_odb10). Repetitive sequence analysis was carried out using RepeatMasker v4.1.2 (Smit *et al*. 2015) in nhmmscan (v3.3.2) mode and the FamDB database, where query species was set as Diptera.

### Hidden Markov model design

Sequences in the final polished genome aligning to the previously characterized PE-degrading enzyme sequences Demetra and Ceres (accession numbers XP_052756923.1 and XP_052756922.1, respectively) were located using the EMBOSS Needle tool (Madeira *et al*. 2022). These sequences, along with the HMMER 2.41.2 software suite from EMBL-EBI (Potter *et al.* 2018), were then utilized to construct a hidden Markov model (HMM) profile for the PE-degrading enzymes. The MPI tool kit (Gabler *et al*. 2020) was used to validate the first HMM generated by HMMER. The GenBank accession numbers for Demetra and Ceres, originally reported by Sanluis-Verdes *et al*. (2022), were then replaced by XP_052756923.1 and XP_052756922.1, respectively, from the polished assembly. The HMM was then used to probe the publicly available reference proteome database in HMMER 2.41.2 for potential homologs to Demetra and Ceres.

### Multiple sequence alignment and phylogenetic analysis

A total of 156 hexamerin homologs detected by HMM probing were aligned using MUSCLE by EMBL-EBI and output as FASTA files (Edgar 2004). Phylogenetic trees were generated using the IQ-TREE2 tool (Nguyen *et al*. 2015; Minh *et al*. 2020) with 1,000 UFBoot bootstrap alignments (Minh *et al*. 2013) and a perturbation strength of 0.5. ModelFinder was used for the best-fit analysis of model selection according to the Bayesian Information Criterion (Kalyaanamoorthy *et al*. 2017). The iTOL server (Letunic and Bork 2021) was used for tree visualization.

### Signal peptide prediction and functional annotation

Secretory proteins in the annotated genome were predicted using SignalP v6 (Teufel *et al*. 2022), with the organism parameter set as Eukaryota. Functional annotations of the predicted secretory proteins were carried out using EggNOG-mapper (Huerta-Cepas *et al*. 2019) with search criteria of a 0.001 minimum *e*-value, 40% identity, and minimum 20% query and subject coverage. The taxonomic scope was set to Arthropoda. Semantic similar gene ontology (GO) terms were removed from the output and offspring terms were summarized to a higher level 2 biological process (BP), molecular function (MF), and cellular component terms with the GOSlim and GSEBase R packages (Wu *et al*. 2021). The enrichGO function of the ClusterProfiler R package was used to identify the proteins with different GO terms. Names and activities of the proteins of interest were extracted from the enzyme-database website (McDonald *et al*. 2009) and manually curated.

### Genome sequence and annotation

This Whole Genome Shotgun project has been deposited at DDBJ/ENA/GenBank under the accession number JAPDED000000000. The version described in this paper is version JAPDED010000000. The genome was annotated using the NCBI Eukaryotic Genome Annotation Pipeline (EGAP; Software version: 10.1). For gene prediction, 423, 40,194, and 9,847 transcript sequences from GenBank, Transcriptome Shotgun Assembly (TSA) database, and expressed sequence tags (EST), respectively, were passed to Gnomon. Additionally, an aggregate of 3,520,505,680 *G. mellonella* RNA-Seq reads originating from the whole organism, fat body, mid gut, antennae, hemocytes, and gut was also used for gene prediction with STAR as part of the EGAP annotation pipeline. The full annotation details are available in the following link: https://www.ncbi.nlm.nih.gov/genome/annotation_euk/Galleria_mellonella/GCF_026898425.1-RS_2022_12/.

## Results and discussion

### Genome assembly and annotation

A total of 52.2 Gb of data from the short- and long-read whole-genome sequencing was generated. Oxford Nanopore sequencing using the PromethION platform accounted for 37.6% of this output, and Illumina 150 bp paired-end sequencing accounted for the remainder. The assembled genome had a total length of 471.9 Mb (N50 = 6.4 Mb) distributed across 221 contigs. This assembly was 99.9% *k*-mer complete with a >40 consensus quality score (QV) according to Polca (MaSURCA v4.0.7; Zimin *et al*. 2013). Based on the protein mode for BUSCO analysis, the assembly was 99.3% [Complete (C): 99.3% (Single-copy (S): 66.7%, Duplicated (D): 32.6%), Fragmented (F): 0.1%, Missing (M): 0.6%, Total number of BUSCOs (n): 5286] and 99.9% [C: 99.9% (S: 75.2%, D: 24.7), F: 0.0%, M: 0.1%, n: 1367] complete using Lepidoptera (lepidoptera_odb10) and insect gene sets (insecta_odb10), respectively. Using the genome mode, the assembly was 98.4% [C: 98.4% (S: 97.4%, D: 1.0%), F: 0.4%, M: 1.2%, n: 5286] and 98.8% [C: 98.8% (S: 98.0%, D: 0.8%), F: 0.3%, M: 0.9%, n: 1367] complete employing the Lepidoptera and insect gene sets (Simão *et al*. 2015), respectively.

Annotation using the NCBI's EGAP predicted a total of 15,138 genes and pseudogenes (13,604 protein-coding and 1,335 noncoding). In total, 23,142 transcripts across these genes were annotated, with multiple transcript variants attributed to 4,342 of the genes. A full genome annotation report is available in the NCBI website (https://www.ncbi.nlm.nih.gov/genome/annotation_euk/Galleria_mellonella/GCF_026898425.1-RS_2022_12/).

Table 1 summarizes improvements in our genome assembly compared with the previous *G. mellonella* RefSeq assembly (Lange *et al*. 2018). Our genome was assembled contiguously rather than via scaffolding, and therefore, a greater proportion of the genome was sequenced with a high level of confidence and was contained within far fewer contigs (40,617 vs 221). Additionally, while similar numbers of genes, protein-coding genes, and pseudogenes were identified, almost 3,000 additional mRNA sequences were identified. The BUSCO scores for our assembly were also substantially higher (99.3 cf. 94.7% for GCA_003640425.2) and indicate a higher-quality genome assembly.

**Table 1.** Comparison of assembly and annotation statistics as well as BUSCO analyses of our current genome assembly (GCA 026898425.1) with the previous RefSeq assembly (GCA 003640425.2) as well as the 2 previous assemblies (GCA 002589825.1 and GCA 004355975.1) for *G. mellonella*.

Statistics	Current assembly	Previous assembly	GCA 002589825.1	GCA 004355975.1
Total size	471.9 Mb	401.9 Mb	578.5 Mb	483.2 Mb
GC content	33.5%	33%	34%	34%
Contigs	221	40,617	1,937	622
N50	6.4 Mb	22.5 kb	952.6 kb	2.9 Mb
L50	25	4,351	154	45
Protein-coding genes	13,604	14,067	N/A	
mRNAs	23,142	20,153		
*BUSCOs* (lepidoptera_odb10) as per NCBI's RefSeq annotation (current 4.1.4; previous 4.0.2)				
Complete	99.3%	94.7%	N/A	
Complete/single-copy	98.1%	93.8%		
Complete/duplicated	1.2%	0.9%		
Fragmented	0.1%	3.2%		
Missing	0.6%	2.1%		
Total	5,286			

The previous 2 assemblies were neither annotated nor were analyzed via BUSCO.

Repetitive sequence analysis of the genome (Table 2) showed that most of its transposable elements were retroelements (1.04%), specifically short interspersed nuclear elements (SINE) (0.01%), long interspersed nuclear elements (LINE) (0.27%), and long terminal repeats (LTR) elements (0.76%), while DNA transposons constituted only 0.12% of the genome. Non-LTR elements had wider and phylogenetically distinct distributions across eukaryotes. Eleven distinct clades dating back to the Precambrian era can be found in Lepidoptera (Malik *et al*. 1999; Tay *et al*. 2010). About 5.55 Mb (1.18%) of interspersed repeats were also observed. Satellites, simple repeats, and low-complexity sequences constituted 0.04, 3.13, and 0.38% of the genome, respectively. Similar percentages of SINE elements were observed across genomes of 4 different lepidopteran species (e.g. *Plutella xylostella*, *Manduca sexta*, *Danaus plexippus*, and *Heliconius melpomene*), but the LINE and LTR percentages varied widely (Zhao *et al*. 2017).

**Table 2.** Repetitive sequence classes in the *G. mellonella* genome.

		No. of elements	Length occupied (bp)	Percentage of sequence
*Retroelements*		*27,327*	*4,888,909*	*1.04*
SINE		587	34,376	0.01
LINE		4,019	1,270,518	0.27
L2/CR1/Rex		342	161,977	0.03
R1/LOA/Jockey		416	89,203	0.02
R2/R4/NeSL		2	125	0
LTR elements		22,721	3,584,015	0.76
BEL/Pao		2,124	1,219,939	0.26
Ty1/Copia		3,496	705,253	0.15
Gypsy/DIRS1		17,101	1,658,823	0.35
*DNA transposons*		*6,904*	*547,927*	*0.12*
hobo-Activator		34	4,032	0
Tc1-IS630-Pogo		5,964	441,259	0.09
PiggyBac		1	48	0
Other		728	85,544	0.02
*Rolling circles*		*38*	*5,132*	*0*
Unclassified		1,839	109,421	0.02
Total interspersed repeats			5,546,257	1.18
Small RNA		254	81,469	0.02
Satellites		2,665	165,553	0.04
Simple repeats		292,397	14,791,978	3.13
Low complexity		36,730	1,773,740	0.38

### Analysis of PE-degrading proteins in *G. mellonella*

This *G. mellonella* genome assembly contains a hexamerin gene (XP_052756922.1) whose inferred amino acid sequence is identical to that of the originally published Ceres gene (XP_026756459.1, now obsolete), another hexamerin gene that corresponds to Cora (XP_026749149.2), and an arylphorin gene (XP_052756923.1) showing 99.6% identify (96% query coverage) to the originally published Demetra gene (XP_026756396.1, now obsolete). These 3 proteins share a relatively high degree of homology at ∼30–32% identity.

Intriguingly, however, a BLASTp search revealed several other proteins with ∼50–66% shared identity (>95% query coverage) to Demetra, Ceres, and Cora in certain other insects that also possess PE catabolic activity (Supplementary Table 1), including another lepidopteran, *Plodia interpunctella*, but also the coleopteran, *Tenebrio molitor* (Brandon *et al*. 2018; Lou *et al*. 2022). This search also recovered homologs in other lepidopterans (e.g. *Ostrinia furnacalis* and *Helicoverpa armigera*) with cellulose-degrading capability but no known PE-degrading activity (Dar *et al*. 2018; Zhang *et al*. 2022) also appeared in the BLASTp results. Given that both cellulose and PE are long-chain polymers, these similarities suggest that these proteins may share features enabling them to bind and catabolize various polymers.

There are also several other, more distantly related, genes encoding potential PE-degrading enzymes in the *G. mellonella* genome. These include a prophenoloxidase (AAK64363.1) and 2 phenoloxidases (XP_052751666.1; XP_026755063.2), which are proteins typically involved in the biosynthesis of melanin, a key component of the immune systems of many insects and plants (González-Santoyo and Córdoba-Aguilar 2012; Blaschek and Pesquet 2021). While distinct from the known PE-degrading hexamerin enzymes, these proteins may potentially exhibit oxidizing and catabolic activity on PE due to their phylogenetic, sequence, and structural similarities (Kayser *et al*. 2009; Liang *et al*. 2019), which can be confirmed through experimental verification. Furthermore, 2 currently unannotated proteins (XP_031768453.1; XP_026758133.1) are highly homologous with laccases from various other insects (up to 65 and 93% sequence identities); laccases are multicopper oxidase enzymes that contribute to PE breakdown in microorganisms, such as *Rhodococcus ruber* and *Rhodococcus opacus* (Goudriaan *et al*. 2023; Zampolli *et al*. 2023).

An orthologous gene comparison between the wax moth and 13 other insect species had previously found that various hydrolases, cytochrome P450s, and enzyme families involved in the oxidation of fatty acids were enriched in number and highly expressed in the wax moth (Kong *et al*. 2019). Specific carboxylesterase, lipase 1 and 3 family enzymes, and fatty acid metabolism-related enzymes were also found to be overexpressed in the fat body of wax moth larvae fed on beeswax, while enzymes involved in the beta oxidation of fatty acids have also been found to be upregulated in the salivary glands of wax moth larvae fed on PE (Peydaei *et al*. 2020). Given the structural similarity of beeswax and PE, it has therefore been suggested that their degradation pathways in wax moth larvae include processes usually associated with beta oxidation of long-chain fatty acids (Kong *et al*. 2019; Peydaei *et al*. 2020).

### Phylogenetic analysis of experimentally validated PE-degrading enzymes

**Fig. 1.**
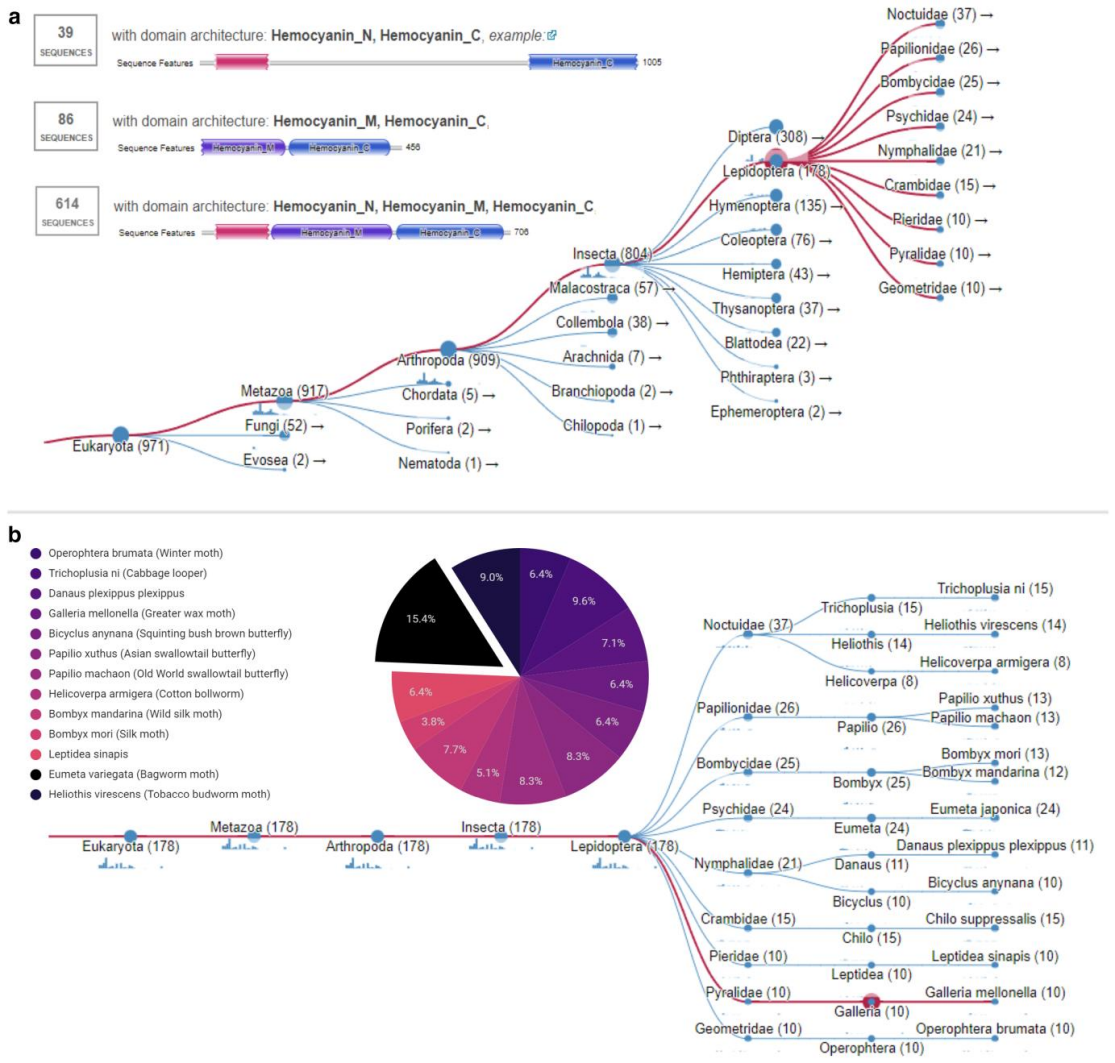
a) The taxonomic distribution of HMM hits across all eukaryotic hits, with clades of Metazoa, Arthropoda, Insecta, and Lepidoptera expanded accordingly. The number of significant hits per node is labeled, and the top 3 PFAM domains conserved across all eukaryotic HMM hits are displayed in the top left corner, with the number of sequence hits per model in text and the location of the domain in the model highlighted. b) The hit distribution from the HMM across species of Lepidoptera. Families are shown and the branch corresponding to *G. mellonella* is highlighted in magenta. The pie chart highlights the corresponding percentage of the HMM hits across the lepidopteran species after factoring out obsolete sequences.

HMMs, established using HMMER 2.41.2 and MPI tool kit, were subsequently constructed to probe publicly available reference genome datasets for potential homologs of Demetra and Ceres. The results showcased a high number of homologous hits for Demetra and Ceres when based on Insecta (82.8% of all Eukaryota hits). Surprisingly, on the order level, analyses returned a higher hit percentage for Diptera (31.7%) than for Lepidoptera (18.3%) (Fig. 1a), despite there being twice the number of sequenced genomes belonging to the latter. Three main conserved protein domains, hemocyanin C (PF03723), hemocyanin M (PF00372), and hemocyanin *N* (PF03722), were found across the whole model, with the *e*-value threshold set to 0.0005, suggesting hits from these HMM are also members of the hemocyanin superfamily.

Seven lepidopteran enzymes exhibited a particularly high match with the HMM and conserved hemocyanin domains, producing an *e*-value of zero and a bit score cut-off of 1,100 (Supplementary Table 2). Three of the 7 enzymes originated from *G. mellonella*, including 2 arylphorins and 1 acidic juvenile hormone-suppressible protein corresponding to Demetra, Cibeles, and Ceres, respectively (Spínola-Amilibia *et al*. 2023). Cora was also recovered using the models, although a nonzero *e*-value of 1E−130 was produced. The remaining 4 enzymes identified may also be potential PE-degrading candidate proteins, particularly as the identities of Cibeles and Cora were found using these HMM prior to the publication of the work by Spínola-Amilibia and co-workers.

Interestingly, the HMM analysis recovered 5 enzymes annotated as phenoloxidases between both the main and validation model tools. Three of these enzymes originated from the coleopteran species *Holotrichia oblita* and 1 each originated from Diptera species *Lucilia cuprina* and *Anopheles moucheti.* However, none of the proteins are lepidopteran in origin despite the HMMER model describing 42 such hits as lepidopteran, a discrepancy which may be the result of different annotation pipelines. Moreover, these results align with existing literature indicating that arthropod hemocyanins serve dual roles as storage proteins and enzymatic facilitators for the breakdown of natural polymers like lignocellulose (Besser *et al.* 2018; Coates and Talbot 2018). This result also supports the potential repurposing of such enzymes for multiple functions (Cubillos *et al*. 2021) and the evolution of hemocyanin domains from a phenoloxidase ancestral sequence (Marieshwari *et al*. 2023).

**Fig. 2.**
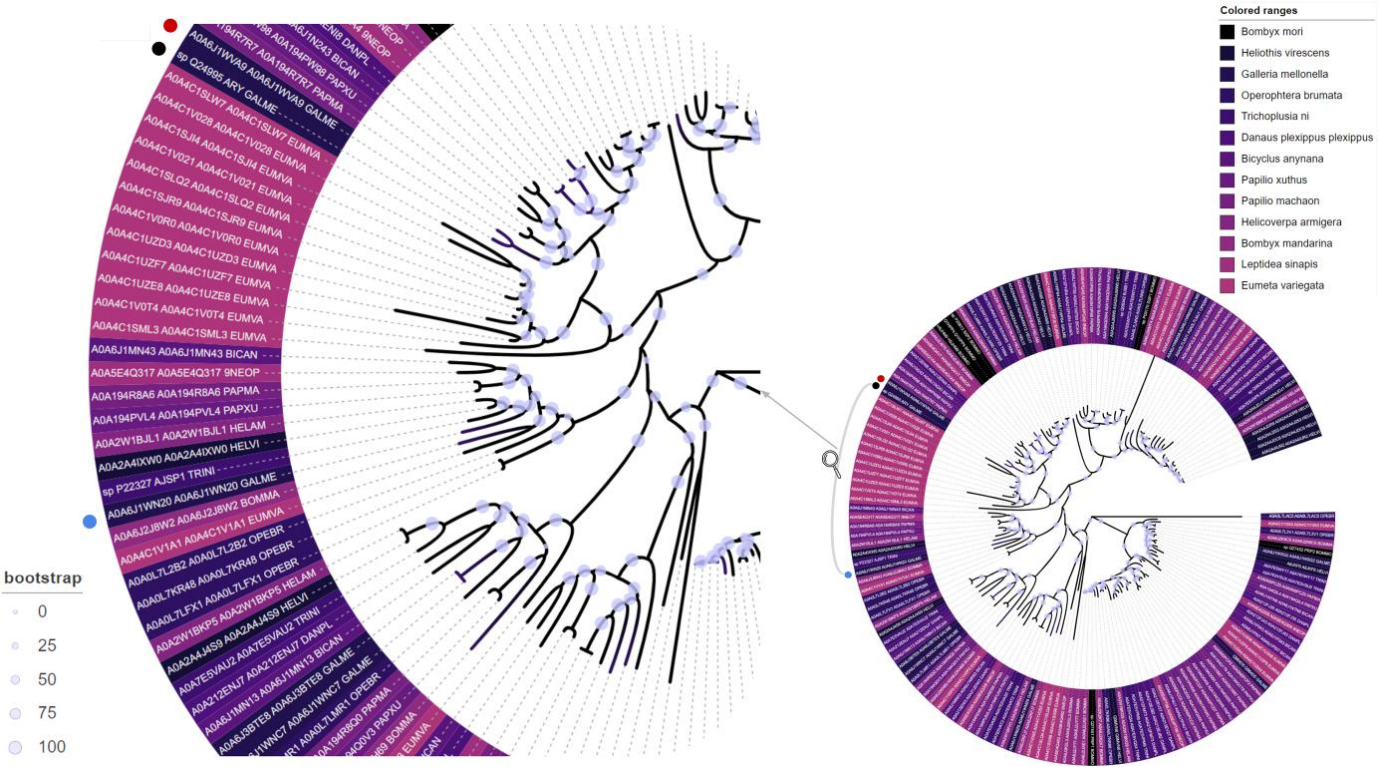
Maximum likelihood phylogenetic tree constructed from the aligned 156 significant hits from HMM iteration on NCBI reference proteomes. The best-fit model was constructed according to the Bayesian information criterion: VT + F + G4. Source species are color coded, and bootstrap values are highlighted according to the legend. Locations of Demetra, Ceres, and Cibeles are marked with red, blue, and black circles, respectively.

A phylogenetic analysis was carried out on the lepidopteran hemocyanin-derived enzyme hits from the HMM for Demetra and Ceres. These enzymes are found in a total of 13 lepidopteran species after removing obsolete sequences (Fig. 2). *Eumeta japonica* had the highest number of such enzymes, followed by *Trichoplusia ni* and *Heliothis virescens* (24, 15, and 14, respectively). Only 10 enzymes were associated with *G. mellonella*, but it was the only 1 of the 3 species in the Pyralidae family with significant hits to this HMM. Six enzymes were associated with *Bombyx mori* (domesticated silk moth), which was the lowest number of model-matching enzymes in the 13 species analyzed, although its wild counterpart (*Bombyx mandarina*) returned 12 matching hits.

Interestingly, phylogenetic analysis reveals that 3 previously characterized PE-degrading enzymes do not cluster together in a single clade. This suggests the possibility of additional PE-degrading enzymes in Lepidoptera. Notably, Demetra occupies different clades in the phylogeny compared with Ceres, while sharing the same branch with Cibeles. Demetra and Ceres are distinct from each other and from 19 other lepidopteran enzymes, which are located in closely adjacent clades. This observation implies that Demetra and Ceres, along with the 19 other enzymes, may possess potential plastic-degrading activity (Fig. 2). Among these 19 enzymes, 12 are from *Eumeta ariegatea*, constituting 50% of all hits from *E. ariegatea* and located between Demetra and Ceres. Notably, an uncharacterized protein from the winter moth *Operophtera brumata* (Uniprot accession no. A0A0L7LAC5) contained both a hemocyanin N and an α/β hydrolase (PFAM ID: PF00561) domain. This protein represents the outgroup sequence in the phylogenetic tree, displaying early divergence from the tree root and is the only protein in the model to contain an α/β hydrolase domain. This domain is found in the majority of plastic-degrading microbial enzymes (Kaushal *et al*. 2021; Buchholz *et al*. 2022), although it was not included in the model input (Demetra and Ceres). Furthermore, a protein (Uniprot accession no. A0A4C1Y3X0), annotated by Protein ANalysis THrough Evolutionary Relationships (PANTHER; a bioinformatic tool containing phylogenetic databases for classifying proteins) (Thomas *et al*. 2022), as PTHR11511:SF4 PHENOLOXIDASE 2-Related, also showed minimal sequence divergence which further supports the evolution of hexamerins from an ancestral phenoloxidase (Marieshwari *et al*. 2023).

### Analysis of secretory proteins

**Fig. 3.**
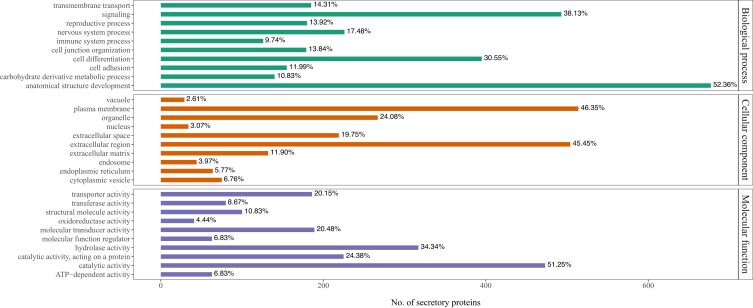
Top 10 GO terms assigned to the identified secretory proteins under each of the biological process, cellular process, and molecular function classifications.

As secreted proteins are more likely to interact with exogenous and insoluble material than intracellular proteins (Feizi *et al*. 2017; Maricchiolo *et al*. 2022), these secreted proteins may have a greater likelihood of exhibiting PE-degrading activity. To identify genes encoding secreted proteins within our genome assembly, we used SignalP v6 (Teufel *et al*. 2022). A total of 3,865 secretory proteins were identified and their potential functions analyzed via an EggNOG-based orthology search (Huerta-Cepas *et al*. 2019) across the Arthropoda phylum. This resulted in functional term assignments for 1,606 proteins, 560 of which are enzymes. Combining the offspring GO terms with parent-level GO terms resulted in 60, 26, and 22 terms for these proteins related to BPs, MF, and/or cellular processes (CPs; Fig. 3).

Of the 1,293 proteins with BP annotations, 52% were related to anatomical structural development, 38% to signaling processes, and 31% to cell differentiation. Notably, however, 11% that were related to carbohydrate-derivative metabolic processes also appeared in the top 10 BP terms, which would be consistent with significant metabolic investment in the enzymatic digestion of complex polymers. Furthermore, of the 1,109 secretory proteins annotated as MF proteins, 51% were predicted to have catalytic activity and 34% to have hydrolase activities. Other prominent MF terms included catalytic activity acting on proteins (24%), transferases (9%), and oxidoreductases (4%). Most of the secreted proteins with CP terms were predicted to be extracellular (45%), or located in the extracellular space (20%), extracellular matrix (12%), or plasma-membrane components (46%).

We then assigned the 560 secreted enzymes to 6 different classes, finding most were hydrolases (342), transferases (109), or oxidoreductases (79), with several also classified as ligases (33), lyases (16), and isomerases (11) (Table 3). The hydrolases and oxidoreductases are of particular interest as members of both classes have been identified as plastic-degrading enzymes (Kaushal *et al*. 2021; Temporiti *et al*. 2022); 135 hydrolases and 10 oxidoreductases in our secretome were predicted to act on ester bonds and peroxide, respectively, and may be potential PE-degrading enzymes in the greater wax moth. Peroxidases are hem-containing oxidoreductases, which are broadly involved in nonspecific oxidation of high redox potential substrates, making these enzymes suitable for diverse industrial and bioremediation usage. Eight percent of the secretory oxidoreductases identified in our *G. mellonella* genome are peroxidases which may be involved in PE degradation via an oxidation pathway.

**Table 3.** Enzyme classes assigned to the identified secretory proteins.

Enzyme classes	No. of secretory proteins
Hydrolases (342)	
Acting on acid anhydrides	6
Acting on carbon–nitrogen bonds, other than peptide bonds	12
Acting on ester bonds (Lipases/Cutinases)^a^	135
Acting on ether bonds	2
Acting on peptide bonds (peptidases)	108
Acting on sulfur–nitrogen bonds	1
Glycosylases	78
Isomerases (11)	
cis-trans-Isomerases	3
Intramolecular oxidoreductases	8
Ligases (3)	
Forming carbon–oxygen bonds	3
Lyases (16)	
Carbon–nitrogen lyases	1
Carbon–oxygen lyases	6
Phosphorus–oxygen lyases	9
Oxidoreductases (79)	
Acting on the CH–CH group of donors	6
Acting on a peroxide as acceptor (peroxidases)^a^	10
Acting on a sulfur group of donors	7
Acting on NADH or NADPH	1
Acting on paired donors, with incorporation or reduction of molecular oxygen	8
Acting on superoxide as acceptor	6
Acting on the aldehyde or oxo group of donors	2
Acting on the CH–NH group of donors	10
Acting on the CH–OH group of donors	28
Oxidizing metal ions	1
Transferases (109)	
Acyltransferases	6
Glycosyltransferases	39
Transferring alkyl or aryl groups, other than methyl groups	2
Transferring 1-carbon groups	1
Transferring phosphorus-containing groups	59
Transferring sulfur-containing groups	2

^a^Potential PE-degrading enzyme candidates.

## Conclusion

We have assembled a RefSeq, contig-level genome of *G. mellonella*. Larvae of the species have been shown to degrade various types of synthetic polymers; hence, it is now attracting considerable academic and industry interest. The mechanism by which the larvae degrade these materials is currently poorly understood, but our analyses of protein-coding sequences retrieved from the new assembly identified several candidate PE-degrading proteins, which will help elucidate the mechanism by which wax moth larvae degrade plastics. Further analysis of these sequences identified a subset of proteins bearing secretory signaling peptides that are more likely to be involved in extracellular activities, such as the degradation of exogenous substances. Additionally, our comparisons of the key Demetra and Ceres proteins with their homologs in other species illustrate the evolutionary history of their component hemocyanin domains, suggesting the potential repurposing of ancestor proteins as oxidoreductases or hydrolases. Evidence of α/β hydrolase domains, a protein domain that is commonly found in many plastic-degrading enzymes, co-occurring with hexamerin enzymes, also suggests an evolutionary relationship.

Following these studies, additional analyses of sex chromosomes using HiC-based chromosome-level scaffolding may reveal sex-specific genes in the wax moth.

## Supplementary Material

jkae070_Supplementary_Data

## Data Availability

The paired-end Illumina and Nanopore sequencing raw data are available as part of NCBI BioProject PRJNA893711. This Whole Genome Shotgun project, along with the Nanopore and Illumina raw data, has been deposited in GenBank under the accession no. JAPDED010000000. The version described in this paper is the first version, JAPDED010000000. Supplemental material available at G3 online.
