# Supplementary material for: Improved reference quality genome sequence of the plastic-degrading greater wax moth, Galleria mellonella
Source: G3 (Bethesda). 2024 Apr 2;14(6):jkae070. doi: 10.1093/g3journal/jkae070 (PMC11152082; doi:10.1093/g3journal/jkae070)
Supplement: jkae070_Supplementary_Data [file jkae070_supplementary_data.pdf]

Improved, reference quality genome sequence of the plastic-degrading greater wax moth, *Galleria mellonella*

Reginald Young[1], Khandaker Asif Ahmed[2], Leon Court[1], Cynthia Castro-Vargas[1], Anna Marcora[3], Joseph Boctor[4], Cate Paull[3], Gene Wijffels[5], Rahul Rane[6], Owain Edwards[7], Tom Walsh[1], Gunjan Pandey[1,*]

[1]CSIRO Environment, Acton, ACT, Australia

[2]CSIRO Australian Animal Health Laboratory, Geelong, VIC, Australia

[3]CSIRO Agriculture and Food, Dutton Park, QLD, Australia

[4]Bioplastics Innovation Hub, Food Futures Institute, Murdoch University, Perth, WA, Australia

[5]CSIRO Agriculture and Food, St. Lucia, QLD, Australia

[6]CSIRO Health and Biosecurity, Parkville, VIC, Australia

[7]CSIRO Environment, Floreat, WA, Australia

*Corresponding author: CSIRO Environment, Acton, ACT, Australia. Email: Gunjan.Pandey@csiro.au

Table S1. Select BLASTp hits for Demetra, Ceres and Cora from organisms with known plastic-degrading or polysaccharide-degrading capabilities.

Query protein	Protein hit	%identity	Accession #
Demetra	Arylphorin-like (*Plodia interpunctella*)	59.72%	XP_053610976.1
	Arylphorin subunit alpha-like (*Ostrinia furnacalis*)	55.89%	XP_028161403.1
	Arylphorin (*Helicoverpa armigera*)	53.71%	AEO51737.1
	Arylphorin subunit alpha-like (*Spodoptera litura*)	52.75%	XP_022822727.1
	Arylphorin 1 (*Tineola bisselliela*)	51.70%	QRN45222.1
Ceres	Acidic juvenile hormone-suppressible protein 1-like (*Ostrinia furnacalis*)	57.81%	XP_028169144.1
	Hexamerine (*Helicoverpa armigera*)	57.65%	AAT76805.1
	Arylphorin 3 (*Tineola bisselliela*)	49.86%	QRN45228.1
Cora	Storage protein (*Ostrinia furnacalis*)	66.05%	ANA05288.1
	Basic juvenile hormone-suppressible protein 1-like (*Ostrinia furnacalis*)	65.78%	XP_028171281.1
	Basic juvenile hormone-suppressible protein 1-like (*Helicoverpa armigera*)	65.53%	XP_021184544.2
	Sex-specific storage protein 1-like (*Plodia interpunctella*)	61.34%	XP_053604600.1

Table S2. The top protein hits matching the developed HMM using the pairwise alignment of Demetra and Ceres and the NCBI reference proteome database as the target. Top hits have an e-value score of zero when compared against the HMM model. The species, bit scores and Uniprot IDs are all displayed accordingly.

Target	Description	Species	Bit Score	E-value
A0A0L7L90_9NEOP	Arylphorin-type storage protein	*Operophtera brumata*	2527.8	0.0e+00
A0A7E5W7Z6_TRINI	Uncharacterized protein LOC113500273	*Trichoplusia ni*	1523.1	0.0e+00
A0A212ENI8_DANPL	Arylphorin-type storage protein	*Danaus plexippus*	1412.5	0.0e+00
A0A6J1WN20_GALME	Acidic juvenile hormone-suppressible protein 1	*Galleria mellonella*	1382.5	0.0e+00
A0A6J1WVA9_GALME	Arylphorin-type storage protein	*Galleria mellonella*	1279.7	0.0e+00
A0A0L7LMJ1_9NEOP	Very-high-density lipoprotein receptor	*Operophtera brumata*	1242.5	0.0e+00
Q24995_GALME	Arylphorin	*Galleria mellonella*	1112.8	0.0e+00
